# Correction: Tamura et al. Interrelations between Gut Microbiota Composition, Nutrient Intake and Diabetes Status in an Adult Japanese Population. *J. Clin. Med.* 2022, *11*, 3216

**DOI:** 10.3390/jcm12010003

**Published:** 2022-12-20

**Authors:** Ayumi Tamura, Masaya Murabayashi, Yuki Nishiya, Satoru Mizushiri, Kiho Hamaura, Ryoma Ito, Shoma Ono, Akihide Terada, Hiroshi Murakami, Jutaro Tanabe, Miyuki Yanagimachi, Itoyo Tokuda, Kaori Sawada, Kazushige Ihara, Makoto Daimon

**Affiliations:** 1 Department of Endocrinology and Metabolism, Hirosaki University Graduate School of Medicine, Hirosaki 036-8562, Japan; 2 Department of Oral Healthcare Science, Hirosaki University Graduate School of Medicine, Hirosaki 036-8562, Japan; 3 Department of Social Medicine, Hirosaki University Graduate School of Medicine, Hirosaki 036-8562, Japan

## Text Correction

There was an error in the original publication [1]. The description of the methods for analyzing gut microbiota was not described properly. The following sentences in the methods were inaccurate and, thus, should be corrected: “Fecal samples were collected within 3 days prior to the study by using a commercial tube kit (TechnoSuruga Laboratory Co., Ltd., Shizuoka, Japan) and cotton swabs and were stored at 4 °C until the DNA was extracted as previously reported [20,21,22]. The gut microbiota composition was investigated by applying the quantitative real-time polymerase chain reaction (q-PCR) method targeting the V3–V4 region of the prokaryotic 16S rRNA genes, as previously described [23].”

A correction was applied to ***2. Subjects and Methods*, *2.2. Characteristics Measured***, ***1st Paragraph, lines 2–7***:

Fecal samples were collected within 3 days prior to the study by using a commercial tube kit (TechnoSuruga Laboratory Co., Ltd., Shizuoka, Japan), and stored at 4 °C until the DNA was extracted, as previously reported [20,21,22]. The gut microbiota composition was investigated by conducting a next-generation sequencing analysis targeting the V3–V4 region of the prokaryotic 16S rRNA genes, as previously described [23].

The authors apologize for any inconvenience caused and state that the scientific conclusions are unaffected. This correction was approved by the Academic Editor. The original publication has also been updated.
